# Supplementary material for: In Silico Searching for Alternative Lead Compounds to Treat Type 2 Diabetes through a QSAR and Molecular Dynamics Study
Source: Pharmaceutics. 2022 Jan 19;14(2):232. doi: 10.3390/pharmaceutics14020232 (PMC8879932; doi:10.3390/pharmaceutics14020232)
Supplement: Supplementary file 1 [file pharmaceutics-14-00232-s001.zip › pharmaceutics-1491706-supplementary.pdf]

pharmaceutics

Supplementary Materials: In Silico Searching for Alternative Lead Compounds to Treat Type 2 Diabetes through a QSAR and Molecular Dynamics Study

Nicolás Cabrera, Sebastián A. Cuesta, José R. Mora, Luis Calle, Edgar A. Márquez, Roland Kaunas and José Luis Paz

Table S1. Name of the molecule, SMILES, pEC_{50} value, docking scores, and training/set label.

Name	SMILES	pEC_{50}	Docking score (kcal/mol)	Label
Molecule1	c1cc(ccc1C#Cc1cc(ccc1)O)OCC(=O)O	5.25	-9.5	Test
Molecule2	c1cc(ccc1C#Cc1c(cccc1)C)OCC(=O)O	5.43	-9.7	Training
Molecule3	c1cc(ccc1C#Cc1ccccc1)OCC(=O)O	5.90	-9.4	Test
Molecule4	c1cc(ccc1C#Cc1cc(ccc1)N)CCC(=O)O	5.96	-9.8	Test
Molecule5	c1cc(ccc1C#Cc1cc(ccc1)CO)CCC(=O)O	6.05	-9.8	Test
Molecule6	c1cc(ccc1C#Cc1cc(ccc1)C)OCC(=O)O	6.29	-9.8	Training
Molecule7	c1cc(ccc1C#Cc1cc(ccc1)C=O)CCC(=O)O	6.45	-9.9	Training
Molecule8	c1cc(ccc1C#Cc1cc(ccc1)C#C)CCC(=O)O	6.81	-10.5	Test
Molecule9	c1cc(ccc1C#Cc1ccc(cc1)C)CCC(=O)O	6.97	-10.0	Test
Molecule10	c1cc(ccc1C#Cc1cc(ccc1)C#N)CCC(=O)O	7.04	-10.3	Test
Molecule11	c1cc(ccc1C#Cc1cc(ccc1)C(F)(F)F)CCC(=O)O	7.05	-10.5	Training
Molecule12	c1cc(ccc1C#Cc1cccc(c1C)C)CCC(=O)O	7.06	-10.5	Training
Molecule13	c1cc(ccc1C#Cc1cccc(c1/C=C/C=C)/C=C/C=C)CCC(=O)O	7.07	-9.3	Training
Molecule14	c1cc(ccc1C#Cc1cc(cc(c1)C)C)CCC(=O)O	7.13	-10.5	Training
Molecule15	c1cc(ccc1C#Cc1c(C)cccc1)CCC(=O)O	7.49	-10.2	Training
Molecule16	c1cc(ccc1C#Cc1ccccn1)CCC(=O)O	4.88	-9.5	Test
Molecule17	c1cc(ccc1C#Cc1ccnc(n1)OC)CCC(=O)O	5.02	-9.3	Training
Molecule18	c1cc(ccc1C#Cc1ccnc(Cl)n1)CCC(=O)O	5.04	-9.6	Training
Molecule19	c1cc(ccc1C#Cc1cccc(n1)C)CCC(=O)O	5.21	-9.9	Training
Molecule20	c1cc(ccc1C#Cc1cccnc1)CCC(=O)O	5.22	-9.6	Training
Molecule21	c1cc(ccc1C#Cc1c(cnc(n1)Cl)C)CCC(=O)O	5.37	-9.7	Training
Molecule22	c1cc(ccc1C#Cc1ccncc1)CCC(=O)O	5.39	-9.6	Training
Molecule23	c1cc(ccc1C#Cc1cncs1)CCC(=O)O	5.41	-8.8	Test
Molecule24	c1cc(ccc1C#Cc1nc(nc(C)c1)Cl)CCC(=O)O	5.56	-9.9	Training
Molecule25	c1cc(ccc1C#Cc1ccnc(C)c1)CCC(=O)O	5.95	-9.9	Training
Molecule26	c1cc(ccc1C#Cc1ccnc(c1)c1c(cccc1)C)CCC(=O)O	5.97	-11.0	Training
Molecule27	c1cc(ccc1C#Cc1cnc(cc1)Cl)CCC(=O)O	6.15	-9.7	Test
Molecule28	c1cc(ccc1C#Cc1cc(cnc1F)C)CCC(=O)O	6.18	-10.3	Training
Molecule29	c1cc(ccc1C#Cc1c(nccc1)Cl)CCC(=O)O	6.22	-10.0	Training
Molecule30	c1cc(ccc1C#Cc1ccnc(c1)OC)CCC(=O)O	6.28	-9.6	Training
Molecule31	c1cc(ccc1C#Cc1ccnc(c1)F)CCC(=O)O	6.29	-9.9	Training
Molecule32	c1cc(ccc1C#Cc1ccnc(c1)Oc1ccccc1)CCC(=O)O	6.32	-10.1	Training
Molecule33	c1cc(ccc1C#Cc1ccnc(c1CC=C)Cl)CCC(=O)O	6.50	-9.2	Training
Molecule34	c1cc(ccc1C#Cc1ccnc(c1)Cl)CCC(=O)O	6.53	-9.9	Training
Molecule35	c1cc(ccc1C#Cc1cccs1)CCC(=O)O	6.60	-9.2	Training
Molecule36	c1cc(ccc1C#Cc1ccnc(c1)c1ccccc1)CCC(=O)O	6.73	-10.6	Training
Molecule37	c1cc(ccc1C#Cc1c(cnc(c1)F)C)CCC(=O)O	6.75	-10.2	Training
Molecule38	c1cc(ccc1C#Cc1ccnc(c1C)Cl)CCC(=O)O	6.87	-10.2	Training
Molecule39	c1cc(ccc1C#Cc1ccnc(c1C)F)CCC(=O)O	6.92	-10.1	Test
Molecule40	c1cc(ccc1C#Cc1ccnc2c1ccc(c2)Cl)CCC(=O)O	7.10	-10.8	Training

Molecule41	c1cc(ccc1C#Cc1c(ccs1)C)CCC(=O)O	7.12	-9.5	Training
Molecule42	c1cc(ccc1C#Cc1cc(nc(c1C)Cl)Cl)CCC(=O)O	7.36	-10.4	Training
Molecule43	c1cc(ccc1C#Cc1cc(nc(c1)Cl)Cl)[C@@H]1[C@@H](C(=O)O)C1	7.37	-9.5	Training
Molecule44	c1cc(ccc1C#Cc1cc(nc(c1)Cl)Cl)CCC(=O)O	7.39	-10.2	Training
Molecule45	C(Cc1ccc(cc1F)OCc1cc(ccc1)c1c(cccc1C)C)C(=O)O	7.46	-10.6	Training
Molecule46	C(Cc1ccc(cc1)OCc1cc(ccc1)c1c(cc(cc1C)OCCCS(=O)(=O)C)C)C(=O)O	7.59	-9.4	Test
Molecule47	C(Cc1ccc(cc1F)NCc1cc(ccc1)c1ccccc1CC)C(=O)O	7.63	-10.4	Training
Molecule48	C(Cc1ccc(cc1)NCc1cc(ccc1)c1ccccc1C)C(=O)O	7.73	-10.3	Training
Molecule49	C(Cc1ccc(cc1F)NCc1cc(ccc1)c1c(cccc1C)C)C(=O)O	7.75	-10.6	Test
Molecule50	C(Cc1ccc(cc1)NCc1cc(ccc1)c1c(cc(cc1)OCCCS(=O)(=O)C)C)C(=O)O	7.76	-9.3	Training
Molecule51	C(Cc1ccc(cc1)NCc1cc(ccc1)c1c(cc(cc1C)OCCCS(=O)(=O)C)C)C(=O)O	7.83	-9.6	Training
Molecule52	C(Cc1ccc(cc1F)NCc1cc(ccc1)c1ccccc1C)C(=O)O	8.03	-10.6	Training
Molecule53	C(Cc1ccc(cc1F)NCc1cc(ccc1)c1c(cc(cc1)OCCCS(=O)(=O)C)C)C(=O)O	8.04	-9.7	Training
Molecule54	c1(ccc(cc1)/C=C/C(=O)O)C#Cc1ccccc1O	4.79	-9.2	Training
Molecule55	c1(ccc(cc1)/C=C/C(=O)O)C#Cc1ccccc1	5.03	-9.2	Test
Molecule56	c1(ccc(cc1)/C=C/C(=O)O)C#Cc1ccccc1C	5.64	-9.5	Training
Molecule57	c1(ccc(cc1)/C=C/C(=O)O)C#Cc1ccccc1CCO	5.69	-9.1	Training
Molecule58	c1(ccc(cc1)CCCC(=O)O)C#Cc1ccccc1	6.00	-10.0	Test
Molecule59	c1(ccc(cc1)/C=C/C(=O)O)C#Cc1ccccc1c1ccccc1	6.00	-9.5	Training
Molecule60	c1(ccc(cc1)CC(=O)O)C#Cc1ccccc1	6.01	-9.9	Training
Molecule61	c1(ccc(cc1)/C=C/C(=O)O)C#Cc1cccc(c1)OCCCS(=O)(=O)C	6.09	-8.5	Training
Molecule62	c1(ccc(cc1)/C=C/C(=O)O)C#Cc1ccccc1CO	6.24	-9.1	Training
Molecule63	c1(ccc(cc1)/C=C/C(=O)O)C#Cc1ccc(cc1)F	6.28	-9.3	Test
Molecule64	c1(ccc(cc1)/C=C/C(=O)O)C#Cc1cc(ccc1C#N)C	6.33	-9.4	Training
Molecule65	c1(ccc(cc1)/C=C/C(=O)O)C#Cc1cccc(c1)O	6.35	-9.2	Training
Molecule66	c1(ccc(cc1)/C=C/C(=O)O)C#Cc1cccc(c1)OCCCCS(=O)(=O)C	6.37	-8.1	Training
Molecule67	c1(ccc(cc1)/C=C/C(=O)O)C#Cc1c(cccc1C)C	6.38	-9.2	Test
Molecule68	c1cc2c(cc1C#Cc1ccccc1)CC[C@H]2CC(=O)O	6.46	-10.2	Training
Molecule69	c1(ccc(cc1)/C=C/C(=O)O)C#Cc1ccccc1C(F)(F)F	6.47	-9.7	Test
Molecule70	c1(ccc(cc1)[C@H]1C[C@@H]1C(=O)O)C#Cc1ccc(cc1)C	6.51	-9.6	Training
Molecule71	c1(ccc(cc1)/C=C/C(=O)O)C#Cc1cccc(c1)OCC#N	6.58	-9.6	Test
Molecule72	c1(ccc(cc1)/C=C/C(=O)O)C#Cc1ccccc1OC(F)(F)F	6.65	-9.5	Training
Molecule73	c1(ccc(cc1)/C=C/C(=O)O)C#Cc1ccccc1OC	6.66	-9.1	Training
Molecule74	c1(ccc(cc1)CCC(=O)O)C#Cc1ccccc1	6.70	-9.9	Test
Molecule75	c1(ccc(cc1)[C@@H](CC(=O)O)C)C#Cc1ccccc1	6.70	-10.4	Training
Molecule76	c1(ccc(cc1)/C=C/C(=O)O)C#Cc1cccc(CC#N)c1	6.72	-10.0	Training
Molecule77	c1(ccc(cc1)/C=C/C(=O)O)C#Cc1ccccc1C(=O)C	6.73	-9.1	Test
Molecule78	c1(ccc(cc1)/C=C/C(=O)O)C#Cc1ccccc1Cl	6.82	-9.3	Training
Molecule79	c1(ccc(cc1)[C@H]1C[C@@H]1C(=O)O)C#Cc1ccccc1	6.84	-9.4	Training
Molecule80	c1(ccc(cc1)[C@H]1C[C@@H]1C(=O)O)C#Cc1c(cccc1)C	6.86	-9.8	Training
Molecule81	c1(ccc(cc1)/C=C/C(=O)O)C#Cc1ccc(cc1C)Cl	6.86	-9.5	Training
Molecule82	c1(ccc(cc1)/C=C/C(=O)O)C#Cc1cc(ccc1C)C	6.91	-9.7	Training
Molecule83	c1(ccc(cc1)/C=C/C(=O)O)C#Cc1cccc(OC(F)(F)F)c1	7.02	-9.8	Training
Molecule84	c1(ccc(cc1)[C@H]1C[C@@H]1C(=O)O)C#Cc1cc(ccc1)C	7.03	-10.0	Training
Molecule85	c1(ccc(cc1)/C=C/C(=O)O)C#Cc1ccccc1CC	7.05	-9.6	Training
Molecule86	c1(ccc(cc1)/C=C/C(=O)O)C#Cc1ccccc1Br	7.08	-9.1	Test
Molecule87	c1(ccc(cc1)CCC(=O)O)C#Cc1cccc(c1)C	7.13	-10.2	Training
Molecule88	c1(ccc(cc1)/C=C/C(=O)O)C#Cc1cccc(c1)OC	7.15	-9.3	Test
Molecule89	c1(ccc(cc1)/C=C/C(=O)O)C#Cc1ccccc1OCC#N	7.33	-8.7	Training
Molecule90	c1(ccc(cc1)/C=C/C(=O)O)C#Cc1ccccc1COC	7.39	-8.8	Training
Molecule91	c1(ccc(cc1)/C=C/C(=O)O)C#Cc1cc(ccc1C)C#N	7.40	-9.4	Test
Molecule92	c1(ccc(cc1)/C=C/C(=O)O)C#Cc1cccc(COC)c1	7.42	-9.2	Training
Molecule93	c1(ccc(cc1)[C@H]1C[C@@H]1C(=O)O)C#Cc1ccccc1CC#N	7.45	-10.3	Training

Table S2. Statistics of the whole and split dataset for the best 10 subsets without the consideration of the applicability domain.

		All dataset		Training/Test set				
Source	Model	R^2	$Q_{CV(5\text{-}fold)}^2$	R^2	$Q_{CV(5\text{-}fold)}^2$	Q_{ext}^2	Technique	Attributes
SS_1213	M1	0.847	0.803	0.872	0.812	0.751	Multiple Linear Regression	10
CSE_1393	M2	0.850	0.807	0.843	0.785	0.855	Multiple Linear Regression	11
SS_1213	M3	0.976	0.704	0.970	0.553	0.751	Random Forest	15
SS_1213	M4	0.970	0.667	0.971	0.569	0.700	Random Forest	17
CSE_1393	M5	0.977	0.708	0.972	0.654	0.704	Random Forest	11
CSE_1393	M6	0.984	0.724	0.983	0.680	0.778	Random Forest	15
CSE_1393	M7	0.980	0.669	0.974	0.532	0.774	Random Forest	9
CSE_1393	M8	0.977	0.707	0.980	0.634	0.802	Random Forest	16
CSE_1393	M9	0.974	0.671	0.966	0.522	0.845	Random Forest	12
SS_1213	M10	0.879	0.645	0.795	0.585	0.619	IBK	6

Table S3. Descriptors names of M1 and M2.

M1	
**Descriptor name**	**Abbreviation**
S_F_AB_nCi_2_M1_SS1_T_KA_psa_MID	X_{3D1}
TS[7]_I50_F_AB_nCi_2_M15_MP3_T_KA_v_MID	X_{3D2}
TS[1]_VC_B_AB_nCi_2_M1_MP0_C_LGL[2-3]_c-h_MID	X_{3D3}
TS[1]_RA_Q_AB_nCi_2_M11_SS6_o_T_KA_v_MID	X_{3D4}
AC[4]_K_F_AB_nCi_2_M8_SS12_o_A_KA_a_MID	X_{3D5}
Q3_B_AB_nCi_2_M14_SS12_A_KA_v-p_MID	X_{3D6}
RA_F_AB_nCi_2_M1_MP0_T_LGL[8-9]_a_MID	X_{3D7}
Q1_F_AB_nCi_2_M11_SS9_X_KA_a_MID	X_{3D8}
I50_F_AB_nCi_2_M8_NS1_T_KA_a_MID	X_{3D9}
GV[2]_N1_Q_AB_nCi_2_M15_MP1_T_KA_e_MID	X_{3D10}
**M2**	
N2_B_AB_nCi_2_M5_MP12_T_KA_a-v_MID	Y_{3D1}
I50_B_AB_nCi_2_M14_SS1_T_KA_r-p_MID	Y_{3D2}
P2_B_AB_nCi_2_M15_SS1_T_KA_v-e_MID	Y_{3D3}
N1_B_AB_nCi_2_M13_SS0_C_LGL[2-3]_c-h_MID	Y_{3D4}
SD_B_AB_nCi_2_M8_NS1_T_KA_a-r_MID	Y_{3D5}
I50_B_AB_nCi_2_M10_MP0_T_KA_r-p_MID	Y_{3D6}
RA_B_AB_nCi_2_M11_SS8_A_KA_v-p_MID	Y_{3D7}
Q2_B_AB_nCi_2_M5_SS2_T_KA_v-e_MID	Y_{3D8}
N2_B_AB_nCi_2_M10_MP1_A_KA_a-psa_MID	Y_{3D9}
CHOQUET[D;0.5;AO1;0.2]_B_AB_nCi_2_M16_MP4_T_KA_v-e_MID	Y_{3D10}
GOWAWA[0.1;2;ES2-OWA;0.9;2;W-OWA;0.7;0.8]_F_AB_nCi_2_M15_MP4_T_KA_v_MID	Y_{3D11}

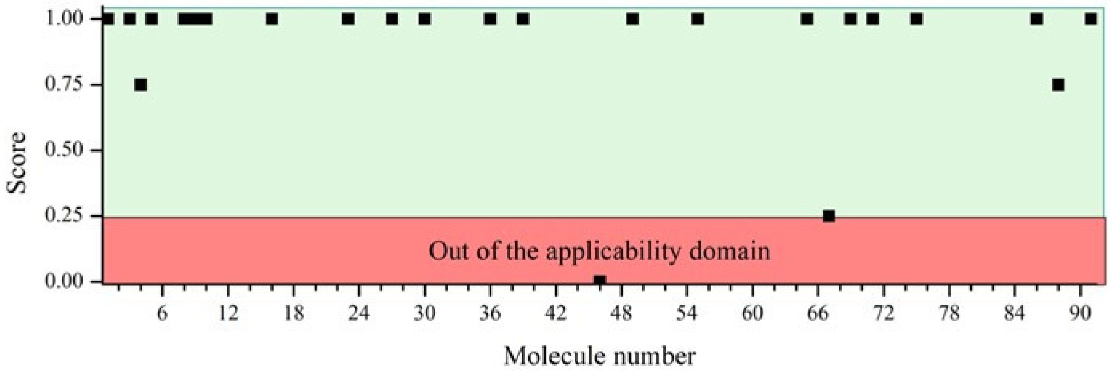

(a)

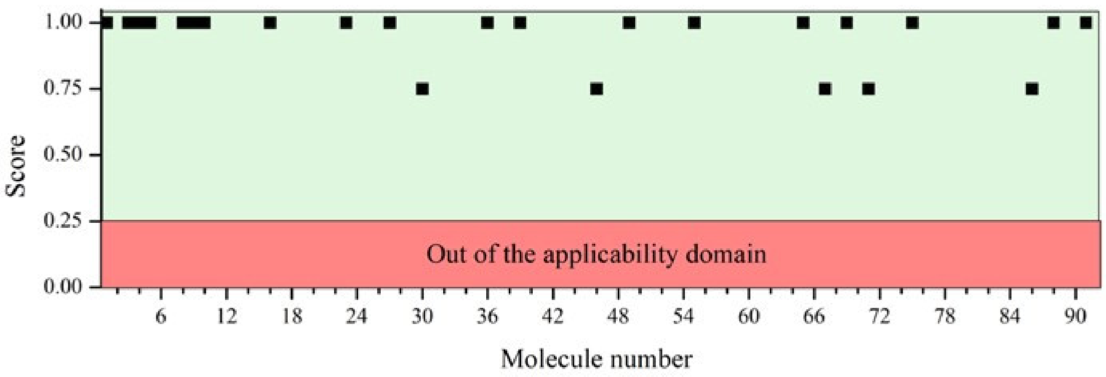

(**b**)

Figure S1. Applicability domain analysis for the test set of M1 (**a**) and M2 (**b**). A compound is considered outlier if the consensus domain score is smaller or equal than 0.25 (red zone).

Table S4. Pearson's coefficient for M1 descriptors.

	X_{3D1}	X_{3D2}	X_{3D3}	X_{3D4}	X_{3D5}	X_{3D6}	X_{3D7}	X_{3D8}	X_{3D9}	X_{3D10}
X_{3D1}	1.000									
X_{3D2}	-0.016	1.000								
X_{3D3}	-0.022	0.212	1.000							
X_{3D4}	0.226	-0.127	-0.158	1.000						
X_{3D5}	0.110	0.002	-0.057	0.085	1.000					
X_{3D6}	-0.430	-0.152	-0.289	0.085	0.006	1.000				
X_{3D7}	-0.073	0.101	0.051	0.120	-0.057	0.078	1.000			
X_{3D8}	0.387	0.113	0.135	0.003	-0.221	-0.293	0.122	1.000		
X_{3D9}	-0.276	-0.248	-0.299	-0.062	0.183	0.408	-0.148	-0.513	1.000	
X_{3D10}	-0.033	0.031	0.017	0.021	-0.061	0.151	0.149	-0.010	-0.021	1.000

Table S5. Pearson's coefficient for M2 descriptors.

	Y_{3D1}	Y_{3D2}	Y_{3D3}	Y_{3D4}	Y_{3D5}	Y_{3D6}	Y_{3D7}	Y_{3D8}	Y_{3D9}	Y_{3D10}	Y_{3D11}
Y_{3D1}	1.000										
Y_{3D2}	-0.330	1.000									
Y_{3D3}	0.119	-0.148	1.000								
Y_{3D4}	-0.171	0.364	-0.234	1.000							
Y_{3D5}	-0.296	0.447	-0.078	0.409	1.000						
Y_{3D6}	0.237	0.184	-0.234	0.453	0.296	1.000					
Y_{3D7}	0.088	-0.106	0.037	0.108	0.203	0.322	1.000				
Y_{3D8}	0.225	-0.262	0.316	-0.037	0.036	0.134	0.208	1.000			
Y_{3D9}	-0.006	0.153	-0.051	0.257	0.233	0.399	0.416	-0.048	1.000		
Y_{3D10}	-0.007	-0.130	0.058	0.011	-0.055	-0.086	0.059	0.159	-0.019	1.000	
Y_{3D11}	0.148	-0.121	0.144	0.025	-0.282	-0.212	-0.170	-0.094	-0.037	-0.046	1.000

Table S6. Name of DiaNat molecule, pEC_{50} predicted value and SMILES of molecules inside the applicability domain.

Name	pEC$_{50}$	SMILES
Capsaicin	6.247	CC(C)/C=C/CCCCC(=O)NCC1=CC(=C(C=C1)O)OC
Cryptolepine	6.264	CN1C2=CC=CC=C2C=C3C1=C4C=CC=CC4=N3
Masoprocol	6.678	C[C@H](CC1=CC(=C(C=C1)O)O)[C@@H](C)CC2=CC(=C(C=C2)O)O
Moracin M	5.157	C1=CC2=C(C=C1O)OC(=C2)C3=CC(=CC(=C3)O)O
Nuciferine	8.005	CN1CCC2=CC(=C(C3=C2[C@H]1CC4=CC=CC=C43)OC)OC
gamma-mangostin	7.832	OC1=C(C(O)=CC2=C1C(C3=C(C(O)=C(C=C3O2)O)C/C=C(C)\C)=O)C/C=C(C)\C
Nepodin	6.433	OC1=CC=CC2=CC(C)=C(C(C)=O)C(O)=C21
Guggulsterone-E	7.451	O=C1CCC2(C)C(CCC3C2CCC4(C)C3CC(/C4=C([H])/C)=O)=C1
Guggulsterone-Z	6.855	O=C1CCC2(C)C(CCC3C2CCC4(C)C3CC(/C4=C(C)/[H])=O)=C1
Isoliquiritigenin	5.982	OC1=CC(O)=CC=C1C(/C=C/C2=CC=C(O)C=C2)=O
2 _ 4 -Dimethoxy-4-hydroxychalcone	6.824	O=C(/C=C/C1=CC=C(O)C=C1)C2=CC=C(OC)C=C2OC
Magnolol	5.937	OC1=CC=C(C2=CC=C(O)C(CC=C)=C2)C=C1CC=C
Pongamol	6.733	O=C(/C=C(O)/C1=CC=CC=C1)C2=C(OC)C(C=CO3)=C3C=C2
Aegeline	7.231	COC1=CC=C(C(O)CNC(/C=C/C2=CC=CC=C2)=O)C=C1
Licochalcone E	7.438	CC([C@H](C)C1=C(O)C=C(OC)C(/C([H])=C(C(C2=CC=C(O)C=C2)=O)\[H])=C1)=C
2 _4 -Dihydroxy-3 _5 -dimethyl-6 -methoxychalcone	6.665	O=C(/C=C/C1=CC=CC=C1)C2=C(OC)C(C)=C(O)C(C)=C2O
Morolic acid	7.646	O[C@H]1CC[C@@]2(C)C(CC[C@]3(C)C2CCC4[C@@]3(C)CCC5(C(O)=O)C4=CC(C)(C)CC5)C1(C)C
Mahanine	6.785	*OC1=CC(NC2=C3C=C(C)C4=C2C=C[C@@](C)(CC/C=C(C)/C)O4)=C3C=C1*
Mahanimbine	6.864	CC1=C2C(C=CC(CC/C=C(C)/C)(C)O2)=C3NC4=CC=CC=C4C3=C1
Apigenin	6.028	C1=CC(=CC=C1C2=CC(=O)C3=C(C=C(C=C3O2)O)O)O
Baicalein	6.328	O=C1C=C(C2=CC=CC=C2)OC3=CC(O)=C(O)C(O)=C31
Chrysin	6.392	O=C(C=C(C1=CC=CC=C1)O2)C3=C2C=C(O)C=C3O
Curcumin	7.69	OC1=C(C=C(C=C1)/C=C/C(CC(/C=C/C2=CC(O)=C(C=C2)OC)=O)=O)OC
Resveratrol	5.444	[H]/C(C1=CC=C(O)C=C1)=C([H])\C2=CC(O)=CC(O)=C2
Phloretin	6.041	OC1=C(C(CCC2=CC=C(O)C=C2)=O)C(O)=CC(O)=C1
6-Gingerol	5.946	CCCCC[C@@H](CC(=O)CCC1=CC(=C(C=C1)O)OC)O
Demethoxycurcumin	6.513	O=C(CC(/C=C/C1=CC=C(O)C(OC)=C1)=O)/C=C/C2=CC=C(O)C=C2
Bisdemethoxycurcumin	7.148	O=C(CC(/C=C/C1=CC=C(O)C=C1)=O)/C=C/C2=CC=C(O)C=C2
Ar-turmerone	6.26	CC1=CC=C([C@@H](C)CC(/C=C(C)/C)=O)C=C1
3-O-Acetyloleanolic acid	8.005	CC1(C)[C@@H](OC(C)=O)CC[C@@]2(C)C1CC[C@]3(C)C2CC=C4[C@@]3(C)CC[C@]5(C(O)=O)[C@@]4([H])CC(C)(C)CC5

Table S7. Name of DrugBank 5.1.7 molecules, pEC$_{50}$ predicted value and SMILES of molecules inside the applicability domain.

Name	pEC$_{50}$	SMILES
eicosapentaenoic acid	7.05	C(=C\C/C=C\C/C=C\C/C=C/C/C=C\CCCC(=O)O)\CC
fluvoxamine	6.853	C(c1ccc(/C(=N/OCCN)/CCCCOC)cc1)(F)(F)F
masoprocol	6.524	Oc1cc(C[C@@H]([C@@H](Cc2cc(O)c(O)cc2)C)C)ccc1O
lorazepam	7.555	Clc1cc2C(=N[C@@H](O)C(=O)Nc2cc1)c1c(Cl)cccc1
indecainide	5.988	O=C(N)C1(CCCNC(C)C)c2c(c3c1cccc3)cccc2
troglitazone	8.009	S1[C@@H](Cc2ccc(OC[C@]3(Oc4c(CC3)c(c(O)c(c4C)C)C)C)cc2)C(=O)NC1=O

dofetilide	6.103	S(=O)(=O)(Nc1ccc(CCN(CCOc2ccc(NS(=O)(=O)C)cc2)C)cc1)C
differin	7.564	O(c1c([C@@]23C[C@H]4C[C@@H](C2)C[C@@H](C3)C4)cc(cc1)c1cc2c(cc1)cc(cc2)C(=O)O)C
eletriptan	5.806	c1ccccc1S(=O)(=O)CCc1cc2c(cc1)[nH]cc2C[C@@H]1N(CCC1)C
reboxetine	5.687	CCOc1ccccc1O[C@H](c1ccccc1)[C@H]1CNCCO1
nevirapine	4.057	O=c1[nH]c2c(n(C3CC3)c3ncccc13)nccc2C
diethylstilbestrol	6.225	c1(ccc(cc1)O)/C(=C(\CC)/c1ccc(cc1)O)/CC
clotrimazole	7.874	Clc1c(C(n2ccnc2)(c2ccccc2)c2ccccc2)cccc1
ropinirole	7.012	O=C1Nc2c(c(CCN(CCC)CCC)ccc2)C1
amsacrine	5.728	S(=O)(=O)(Nc1cc(OC)c(Nc2c3c(nc4c2cccc4)cccc3)cc1)C
disopyramide	6.886	n1ccccc1[C@@](CCN(C(C)C)C(C)C)(C(=O)N)c1ccccc1
travoprost	7.647	FC(F)(F)c1cc(OC[C@H](O)/C=C/[C@@H]2[C@H]([C@@H](O)C[C@H]2O)C/C=C\CCCC(=O)OC(C)C)ccc1
atomoxetine	5.959	O([C@H](CCNC)c1ccccc1)c1c(cccc1)C
bexarotene	8.831	OC(=O)c1ccc(C(=C)c2cc3C(CCC(c3cc2C)(C)C)(C)C)cc1
gefitinib	7.57	Clc1cc(Nc2ncnc3c2cc(OCCCN2CCOCC2)c(OC)c3)ccc1F
amitriptyline	7.005	N(CCC=C1c2c(CCc3c1cccc3)cccc2)(C)C
indomethacin	7.438	c1c(ccc(c1)C(=O)n1c2c(c(c1C)CC(=O)O)cc(cc2)OC)Cl
omeprazole	5.883	[S@](=O)(Cc1ncc(c(OC)c1C)C)c1[nH]c2c(n1)ccc(OC)c2
terfenadine	8.382	c1cccc(c1)C(c1ccccc1)([C@@H]1CCN(CC1)CCC[C@@H](c1ccc(cc1)C(C)(C)C)O)O
clobazam	7.807	Clc1cc2N(C(=O)CC(=O)N(c2cc1)C)c1ccccc1
DMAP	8.085	CC1=C[C@@H]2[C@H](CC[C@@]3(C)[C@H]2CC[C@@]3(C(=O)C)OC(=O)C)[C@@]2(C)CCC(=O)C=C12
treprostinil	6.694	c12cccc(c1C[C@@H]1[C@H](C2)[C@H]([C@@H](C1)O)CC[C@H](CCCCC)O)OCC(=O)O
dydrogesterone	6.706	O=C([C@@H]1[C@@]2([C@H]([C@H]3[C@@H](CC2)[C@]2(C(=CC(=O)CC2)C=C3)C)CC1)C)C
progesterone	6.662	O=C([C@@H]1[C@@]2([C@H]([C@H]3[C@@H]([C@@]4(C(=CC(=O)CC4)CC3)C)CC2)CC1)C)C
sorafenib	6.906	Clc1c(cc(NC(=O)Nc2ccc(Oc3cc(ncc3)C(=O)NC)cc2)cc1)C(F)(F)F
neurol	5.917	Clc1cc2c(n3c(nnc3C)CN=C2c2ccccc2)cc1
spironolactone	8.458	S([C@H]1[C@H]2[C@H]3[C@@]([C@@]4(OC(=O)CC4)CC3)(CC[C@@H]2[C@@]2(C(=CC(=O)CC2)C1)C)C)C(=O)C
zolpidem	7.819	O=C(N(C)C)Cc1n2c(nc1c1ccc(cc1)C)ccc(c2)C
triprolidine	6.476	N1(CCCC1)C/C=C(\c1ccc(cc1)C)/c1ncccc1
periactin	6.492	N1(CCC(=C2c3c(C=Cc4c2cccc4)cccc3)CC1)C
cerivastatin	8.678	Fc1ccc(c2c(c(nc(c2/C=C/[C@@H](O)C[C@@H](O)CC(=O)O)C(C)C)C(C)C)COC)cc1
droperidol	7.341	Fc1ccc(C(=O)CCCN2CCC(=CC2)n2c3c([nH]c2=O)cccc3)cc1
acitretin	8.688	c1(c(c(cc(c1/C=C/C(=C/C=C/C(=C/C(=O)O)/C)/C)C)OC)C)C
nabumetone	5.854	O(c1cc2c(cc(CCC(=O)C)cc2)cc1)C
THC	6.478	[C@H]12c3c(OC([C@@H]1CCC(=C2)C)(C)C)cc(cc3O)CCCCC

montelukast	8.788	Clc1cc2nc(/C=C/c3cc([C@H](SCC4(CC4)CC(=O)O)CCc4c(C(O)(C)C)cccc4)ccc3)ccc2cc1
duloxetine	5.676	s1c([C@@H](Oc2c3c(ccc2)cccc3)CCNC)ccc1
raloxifene	7.719	s1c(c(c2c1cc(O)cc2)C(=O)c1ccc(OCCN2CCCCC2)cc1)c1ccc(O)cc1
nabilone	7.053	O1C([C@H]2[C@@H](CC(=O)CC2)c2c1cc(C(CCCCCC)(C)C)cc2O)(C)C
darifenacin	8.078	O=C(N)C([C@@H]1CCN(C1)CCc1cc2c(OCC2)cc1)(c1ccccc1)c1ccccc1
tolmetin	6.532	O=C(c1n(c(cc1)CC(=O)O)C)c1ccc(cc1)C
haloperidol	8.982	Clc1ccc(C2(O)CCN(CC2)CCCC(=O)c2ccc(F)cc2)cc1
carteolol	5.305	O(C[C@H](O)CNC(C)(C)C)c1c2CCC(=O)Nc2ccc1
alitretinoin	6.885	OC(=O)/C=C(/C=C/C=C(\C=C\C1=C(CCCC1(C)C)C)/C)\C
dibucaine	6.933	O(c1nc2c(c(c1)C(=O)NCCN(CC)CC)cccc2)CCCC
rofecoxib	7.353	S(=O)(=O)(c1ccc(C2=C(c3ccccc3)C(=O)OC2)cc1)C
toremifene	8.58	ClCC/C(=C(/c1ccc(OCCN(C)C)cc1)\c1ccccc1)/c1ccccc1
nortriptyline	5.793	N(CCC=C1c2c(CCc3c1cccc3)cccc2)C
amoxapine	7.118	Clc1cc2C(=Nc3c(Oc2cc1)cccc3)N1CCNCC1
carbamazepine	4.992	O=C(N1c2c(C=Cc3c1cccc3)cccc2)N
cinnarizine	8.049	N1(CCN(CC1)CC=Cc1ccccc1)C(c1ccccc1)c1ccccc1
propranolol	5.306	c1ccc(c2c1cccc2)OC[C@@H](CNC(C)C)O
fenoprofen	7.224	O(c1cc([C@H](C)C(=O)O)ccc1)c1ccccc1
voriconazole	5.577	Fc1c([C@](O)([C@@H](C)c2ncncc2F)Cn2ncnc2)ccc(F)c1
{2-[(2,6-dichloro-3,4-dihydroxyphenyl)amino]phenyl}acetic acid	6.587	Clc1c(Nc2c(CC(=O)O)cccc2)c(Cl)ccc1
labetalol	6.09	O[C@H](CN[C@H](CCc1ccccc1)C)c1cc(c(O)cc1)C(=O)N
provera	8.445	C1C[C@]([C@]2(CC[C@@H]3[C@@]4(C(=CC(=O)CC4)[C@H](C[C@H]3[C@H]12)C)C)C)(OC(=O)C)C(=O)C
sulindac	7.536	C\1(=C\c2ccc(cc2)[S@@](=O)C)/C(=C(c2c1ccc(c2)F)CC(=O)O)C
6-bromo-4-[(dimethylamino)methyl]-3-(ethoxycarbonyl)-2-[(phenylsulfanyl)methyl]-1H-indol-5-yl sulfate	6.926	n1ccc(c2c1cc(cc2)Cl)N[C@H](CCCN(CC)CC)C
amodiaquine	6.673	Clc1cc2nccc(Nc3cc(CN(CC)CC)c(O)cc3)c2cc1
astemizole	7.712	Fc1ccc(Cn2c(NC3CCN(CC3)CCc3ccc(OC)cc3)nc3c2cccc3)cc1
dyclonine	6.624	O=C(CCN1CCCCC1)c1ccc(OCCCC)cc1
latanoprost	6.835	[C@H]1([C@@H]([C@H]([C@@H](C1)O)CC[C@@H](O)CCc1ccccc1)C/C=C/CCCC(=O)OC(C)C)O
estrone	6.941	O=C1[C@@]2([C@H]([C@H]3[C@H](CC2)c2c(CC3)cc(O)cc2)CC1)C
tamoxifen	8.327	O(c1ccc(/C(=C(/CC)\c2ccccc2)/c2ccccc2)cc1)CCN(C)C
losartan	6.195	Clc1nc(n(Cc2ccc(cc2)c2c(cccc2)c2n[nH]nn2)c1CO)CCCC
midazolam	6.41	Clc1cc2c(n3c(CN=C2c2c(F)cccc2)cnc3C)cc1
flurazepam	7.257	Clc1cc2c(N(CCN(CC)CC)C(=O)CN=C2c2c(F)cccc2)cc1
phentolamine	6.251	Oc1cc(N(CC2=NCCN2)c2ccc(cc2)C)ccc1
fluorescein	7.147	Oc1ccc2C3(c4c(Oc2c1)cc(cc4)O)OC(=O)c1c3cccc1

delavirdine	6.712	S(=O)(=O)(Nc1cc2cc([nH]c2cc1)C(=O)N1CCN(CC1)c1ncccc1NC(C)C)C
tamsulosin	5.105	S(=O)(=O)(N)c1cc(C[C@H](NCCOc2c(OCC)cccc2)C)ccc1OC
flurbiprofen	6.629	Fc1c(ccc([C@H](C)C(=O)O)c1)c1ccccc1
apomorphine	6.242	Oc1c2c3c4[C@H](N(CCc4ccc3)C)Cc2ccc1O
paroxetine	7.548	Fc1ccc([C@H]2[C@@H](CNCC2)COc2cc3OCOc3cc2)cc1
(1S,2S,4R,8S,9S,11S,12R,13S)-12-fluoro-11-hydroxy-8-(2-hydroxyacetyl)-6,6,9,13-tetramethyl-5,7-dioxapentacyclo[10.8.0.0^{2,9}.0^{4,8}.0^{13,18}]icosa-14,17-dien-16-one	5.142	c12c(cccc1)nc(c1c2n(cn1)CC(C)C)N
naftin	8.162	N(Cc1c2c(ccc1)cccc2)(CC=Cc1ccccc1)C
pentamidine	6.204	O(CCCCCOc1ccc(cc1)C(=N)N)c1ccc(cc1)C(=N)N
modafinil	5.468	[S@@](=O)(C(c1ccccc1)c1ccccc1)CC(=O)N
(R)-6-hydroxywarfarin	8.378	C1(CCCC(=C1/C=C/C(=C/C=C/C(=C/C(=O)O)/C)/C)C)(C)C
patanol	8.028	O1c2c(/C(=C\CCN(C)C)/c3c(C1)cccc3)cc(cc2)CC(=O)O
tirofiban	6.765	S(=O)(=O)(N[C@@H](Cc1ccc(OCCCCC2CCNCC2)cc1)C(=O)O)CCCC
9-oxo-2-azatricyclo[9.4.0.0^{3,8}]pentadeca-1(15),3,5,7,11,13-hexaene-2-carboxamide	5.154	c1ccc2c(c1)CC(=O)c1c(N2C(=O)N)cccc1
mefenamic acid	6.241	OC(=O)c1c(Nc2c(c(ccc2)C)C)cccc1
tazarotene	7.277	S1CCC(c2c1ccc(c2)C#Cc1ncc(cc1)C(=O)OCC)(C)C
fenoldopam	6.215	Clc1c2c([C@H](CNCC2)c2ccc(O)cc2)cc(O)c1O
halazepam	8.288	Clc1cc2c(N(CC(F)(F)F)C(=O)CN=C2c2ccccc2)cc1
proparacaine	6.444	O(CCN(CC)CC)C(=O)c1cc(N)c(OCCC)cc1
fentanyl	6.676	O=C(N(C1CCN(CC1)CCc1ccccc1)c1ccccc1)CC
carprofen	6.648	Clc1cc2c3c([nH]c2cc1)cc([C@@H](C)C(=O)O)cc3
ethynodiol diacetate	6.248	O([C@@]1([C@@]2([C@H]([C@H]3[C@H](CC2)[C@@H]2C(=C[C@@H](OC(=O)C)CC2)CC3)CC1)C)C#C)C(=O)C
diazepam	7.545	Clc1cc2c(N(C(=O)CN=C2c2ccccc2)C)cc1
dobutamine	5.851	Oc1cc(CCN[C@@H](CCc2ccc(O)cc2)C)ccc1O
clofazimina	8.522	c12n(c3c(nc1cc(c(=NC(C)C)c2)Nc1ccc(Cl)cc1)cccc3)c1ccc(cc1)Cl
PrePar	5.681	O[C@@H]([C@@H](NCCc1ccc(O)cc1)C)c1ccc(O)cc1
suprofen	7.583	s1c(C(=O)c2ccc([C@H](C)C(=O)O)cc2)ccc1
vaprisol	6.855	O=C(N1CCc2[nH]c(nc2c2c1cccc2)C)c1ccc(NC(=O)c2c(c3ccccc3)cccc2)cc1
eprosartan	7.779	n1(Cc2ccc(cc2)C(=O)O)c(/C=C(/C(=O)O)\Cc2sccc2)cnc1CCCC
pioner	7.506	c1cccc(c1)C(=C(Cl)c1ccccc1)c1ccc(cc1)OCCN(CC)CC
bumetanide	4.898	S(=O)(=O)(N)c1c(Oc2ccccc2)c(NCCCC)cc(c1)C(=O)O
[1-({2-[({5-[(dimethylamino)methyl]furan-2-yl}methyl)sulfanyl]ethyl}amino)-2-nitroethenyl](methyl)amine	7.477	C/C=C(\c1ccc(cc1)O)/C(=C/C)/c1ccc(cc1)O
oxybuprocaine	6.83	C(=O)(c1cc(c(cc1)N)OCCCC)OCCN(CC)CC

triazolam	6.338	Clc1cc2c(n3c(nnc3C)CN=C2c2c(Cl)cccc2)cc1
3-({3-[(isopropyl-C-hydroxycarbonimidoyl)aminosulfonyl]-1H-pyridin-4-ylidene}amino)benzoic acid	6.545	[C@@H]1(C[C@H]([C@@H]([C@H]1C/C=C\CCCC(=O)NCC)/C=C/[C@H](CCc1ccccc1)O)O)O
repaglinide	7.424	O=C(N[C@H](c1c(N2CCCCC2)cccc1)CC(C)C)Cc1cc(OCC)c(cc1)C(=O)O
paricalcitol	7.817	C1(CCN(CC1)CCc1ccc(N)cc1)(C(=O)OCC)c1ccccc1
cyclobenzaprine	7.254	N(CCC=C1c2c(C=Cc3c1cccc3)cccc2)(C)C
salmeterol	6.414	O(CCCCCCNC[C@@H](O)c1cc(c(O)cc1)CO)CCCCc1ccccc1
meclofenamic acid	7.171	Clc1c(Nc2c(cccc2)C(=O)O)c(Cl)ccc1C
norgestimate	6.618	C1C[C@]([C@]2(CC[C@@H]3[C@@H]4C(=C/C(=N/O)/CC4)CC[C@H]3[C@H]12)CC)(C#C)OC(=O)C
bromfenac	8.469	Brc1ccc(C(=O)c2c(N)c(CC(=O)O)ccc2)cc1
clarinex	6.361	Clc1cc2c(C(=C3CCNCC3)c3ncccc3CC2)cc1
azelastine	8.083	Clc1ccc(Cc2nn([C@@H]3CCCN(CC3)C)c(=O)c3c2cccc3)cc1
ezetimibe	6.956	c1cc(ccc1F)N1C(=O)[C@@H]([C@H]1c1ccc(cc1)O)CC[C@@H](c1ccc(cc1)F)O
isotretinoin	7.461	C1(CCCC(=C1/C=C/C(=C/C=C/C(=C\C(=O)O)/C)/C)C)(C)C
formoterol	6.69	O[C@H](CN[C@H](Cc1ccc(OC)cc1)C)c1cc(NC=O)c(O)cc1
activin	7.264	O([C@@H]1[C@@]2([C@H]([C@H]3[C@H](CC2)[C@@H]2C(=CC(=O)CC2)CC3)CC1)C)C(=O)CCc1ccccc1
oxaprozin	5.196	o1c(c(nc1CCC(=O)O)c1ccccc1)c1ccccc1
(-)-bupivacaine	6.997	O=C(Nc1c(cccc1C)C)[C@H]1N(CCCC1)CCCC
ketoprofen	6.586	OC(=O)[C@H](c1cc(ccc1)C(=O)c1ccccc1)C
cinacalcet	7.172	FC(F)(F)c1cc(CCCN[C@@H](c2c3c(ccc2)cccc3)C)ccc1
vitamin K	9.23	c12C(=O)C(=C(C(=O)c1cccc2)C/C=C(/CCC[C@@H](CCC[C@@H](CCCC(C)C)C)C)\C)C
antara	7.654	Clc1ccc(C(=O)c2ccc(OC(C)(C)C(=O)OC(C)C)cc2)cc1
acetophenazine	7.217	N1(c2c(Sc3c1cccc3)ccc(c2)C(=O)C)CCCN1CCN(CC1)CCO
glipizide	6.549	S(=O)(=O)(NC(=O)NC1CCCCC1)c1ccc(cc1)CCNC(=O)c1ncc(nc1)C
atorvastatin	8.59	Fc1ccc(c2n(CC[C@@H](O)C[C@@H](O)CC(=O)O)c(C(C)C)c(C(=O)Nc3ccccc3)c2c2ccccc2)cc1
butenafine	8.511	N(Cc1ccc(C(C)(C)C)cc1)(Cc1c2c(ccc1)cccc2)C
fluvastatin	7.116	Fc1ccc(c2c(n(C(C)C)c3c2cccc3)/C=C/[C@H](O)C[C@H](O)CC(=O)O)cc1
pimozide	7.446	Fc1ccc(C(CCCN2CCC(n3c4c([nH]c3=O)cccc4)CC2)c2ccc(F)cc2)cc1
arbutamine	5.349	O[C@@H](CNCCCCc1ccc(O)cc1)c1cc(O)c(O)cc1
papaverine	6.889	O(c1cc2c(nccc2cc1OC)Cc1cc(OC)c(OC)cc1)C
proflavine	4.232	n1c2c(cc3c1cc(N)cc3)ccc(N)c2
carvedilol	6.047	c1(c2c(ccc1)[nH]c1c2cccc1)OC[C@@H](CNC-COc1ccccc1OC)O
sulfinpyrazone	7.554	[S@@](=O)(CCC1C(=O)N(N(C1=O)c1ccccc1)c1ccccc1)c1ccccc1
desipramine	5.781	N1(c2c(CCc3c1cccc3)cccc2)CCCNC
propafenone	6.646	c1(C(=O)CCc2ccccc2)c(OC[C@H](O)CNCCC)cccc1
acebutolol	6.301	c1(c(cc(cc1)NC(=O)CCC)C(=O)C)OC[C@@H](CNC(C)C)O

akÃŽÂ²	6.431	O(c1c2CCCC(=O)c2ccc1)C[C@@H](O)CNC(C)(C)C
estazolam	5.888	Clc1cc2c(n3c(nnc3)CN=C2c2ccccc2)cc1
levomethadyl acetate	7.594	O([C@H](C(C[C@@H](N(C)C)C)(c1ccccc1)c1ccccc1)CC)C(=O)C
encainide	6.955	C(=O)(Nc1c(CC[C@@H]2N(C)CCCC2)cccc1)c1ccc(cc1)OC
lapatinib	7.16	Clc1cc(Nc2ncnc3c2cc(c2oc(CNCCS(=O)(=O)C)cc2)cc3)ccc1OCc1cc(F)ccc1
sunitinib	6.965	Fc1cc2/C(=C/c3[nH]c(c(c3C)C(=O)NCCN(CC)CC)C)/C(=O)Nc2cc1
arformoterol	6.749	O[C@@H](CN[C@@H](Cc1ccc(OC)cc1)C)c1cc(NC=O)c(O)cc1
lumiracoxib	5.866	c1(c(cccc1Cl)F)Nc1ccc(cc1CC(=O)O)C
fenoterol	6.276	O[C@@H](CN[C@H](Cc1ccc(O)cc1)C)c1cc(O)cc(O)c1
Pro-Air	6.385	O[C@@H]([C@@H](NC(C)C)CC)c1c2c([nH]c(=O)cc2)c(O)cc1
yasmin	7.614	O1[C@]2([C@@H]3[C@H]([C@@H]4[C@@]2(CC[C@H]2[C@H]4[C@@H]4[C@@H](C4)C4=CC(=O)CC[C@]24C)C)C3)CCC1=O
oxybenzone	6.888	O(c1cc(O)c(C(=O)c2ccccc2)cc1)C
almitrine	7.607	Fc1ccc(C(N2CCN(CC2)c2nc(nc(n2)NCC=C)NCC=C)c2ccc(F)cc2)cc1
bromazepam	6.539	Brc1cc2c(NC(=O)CN=C2c2ncccc2)cc1
prazepam	7.571	Clc1cc2c(N(CC3CC3)C(=O)CN=C2c2ccccc2)cc1
propericiazine	8.311	S1c2c(N(CCCN3CCC(O)CC3)c3c1cccc3)cc(cc2)C#N
deferasirox	5.517	n1c(nn(c1c1ccccc1O)c1ccc(cc1)C(=O)O)c1ccccc1O
phenindamine	6.834	N1(CC2=C(CC1)c1c([C@@H]2c2ccccc2)cccc1)C
etoricoxib	6.428	Clc1cc(c2ccc(S(=O)(=O)C)cc2)c(nc1)c1ccc(nc1)C
piretanide	5.19	NS(=O)(=O)c1cc(cc(N2CCCC2)c1Oc1ccccc1)C(=O)O
docosahexaenoic acid	6.331	C(=C\C/C=C\C/C=C\C/C=C\C/C=C\C/C=C\CCC(=O)O)\CC
Ogen	5.879	C1CC(=O)[C@]2(CC[C@@H]3c4c(CC[C@H]3[C@H]12)cc(cc4)OS(=O)(=O)O)C
bifonazole	6.257	n1([C@@H](c2ccc(cc2)c2ccccc2)c2ccccc2)ccnc1
phenolphthalein	7.505	O1C(c2c(C1=O)cccc2)(c1ccc(O)cc1)c1ccc(O)cc1
prenylamine	6.388	N(CCC(c1ccccc1)c1ccccc1)[C@@H](Cc1ccccc1)C
4,4-difluoro-N-[(1S)-3-[(1R,3S,5S)-3-(3-isopropyl-5-methyl-1,2,4-triazol-4-yl)-8-azabicyclo[3.2.1]octan-8-yl]-1-phenylpropyl]cyclohexane-1-carboxamide	8.202	N1(CC[C@H](NC(=O)C2CCC(CC2)(F)F)c2ccccc2)[C@H]2C[C@H](C[C@@H]1CC2)n1c(nnc1C(C)C)C
flunarizine	8.214	Fc1ccc(C(N2CCN(CC2)C/C=C/c2ccccc2)c2ccc(F)cc2)cc1
multaq	7.423	S(=O)(=O)(Nc1cc2c(c(oc2cc1)CCCC)C(=O)c1ccc(OCCCN(CCCC)CCCC)cc1)C
nilotinib	6.834	c1c(cc(cc1NC(=O)c1ccc(c(c1)Nc1nccc(n1)c1cnccc1)C)C(F)(F)F)n1cnc(c1)C
talion	7.725	Clc1ccc([C@H](OC2CCN(CC2)CCCC(=O)O)c2ncccc2)cc1
ospemifene	7.392	ClCC/C(=C(/c1ccc(OCCO)cc1)\c1ccccc1)/c1ccccc1
iloperidone	6.614	Fc1cc2onc(C3CCN(CC3)CCCOc3c(OC)cc(cc3)C(=O)C)c2cc1
retigabine	5.721	Fc1ccc(CNc2cc(N)c(NC(=O)OCC)cc2)cc1
indacaterol	7.408	O[C@@H](CNC1Cc2c(C1)cc(c(c2)CC)CC)c1c2c([nH]c(=O)cc2)c(O)cc1

vandetanib	8.331	c12c(Nc3c(cc(cc3)Br)F)ncnc1cc(c(c2)OC)OCC1CCN(CC1)C
abiraterone	5.613	C1[C@@H](CC2=CC[C@@H]3[C@@H]([C@]2(C1)C)CC[C@]1(C)C(=CC[C@@H]31)c1cnccc1)O
nylidrin	5.895	C[C@@H](CCc1ccccc1)N[C@H](C)[C@H](c1ccc(cc1)O)O
lasofoxifene	6.706	c1ccc(cc1)[C@H]1CCc2cc(ccc2[C@H]1c1ccc(cc1)OCCN1CCCC1)O
prasugrel	8.59	CC(=O)Oc1cc2c(CCN(C2)[C@@H](C(=O)C2CC2)c2ccccc2F)s1
arzoxifene	8.218	COc1ccc(cc1)c1c(c2c(s1)cc(cc2)O)Oc1ccc(cc1)OCCN1CCCCC1
modafinil	5.985	C(c1ccccc1)(c1ccccc1)[S@](=O)CC(=O)N
agomelatine	4.923	O(c1cc2c(CCNC(=O)C)cccc2cc1)C
edaglitazone	6.937	c1(ccc(cc1)/C=C/C(=O)NO)CNCCc1c([nH]c2c1cccc2)C
xylometazoline	6.963	Cc1cc(cc(C)c1CC1=NCCN1)C(C)(C)C
fesoterodine	8.206	CC(C)C(=O)Oc1c(cc(cc1)CO)[C@H](CCN(C(C)C)C(C)C)c1ccccc1
aceclofenac	6.931	c1ccc(c(c1)CC(=O)OCC(=O)O)Nc1c(cccc1Cl)Cl
drotaverine	5.75	O(c1cc2CCN/C(=C\c3cc(OCC)c(OCC)cc3)/c2cc1OCC)CC
capsaicin	6.335	O=C(NCc1cc(OC)c(O)cc1)CCCC/C=C/C(C)C
nepafenac	5.613	c1ccc(cc1)C(=O)c1c(c(ccc1)CC(=O)N)N
tebamin	6.152	c1ccc(cc1)OC(=O)c1ccc(cc1O)N
protokylol	5.816	C[C@@H](Cc1ccc2OCOc2c1)NC[C@@H](O)c1ccc(O)c(O)c1
triclosan	7.106	O(c1c(cc(cc1)Cl)Cl)c1c(cc(cc1)Cl)O
antazoline	6.408	N(CC1=NCCN1)(Cc1ccccc1)c1ccccc1
dimethindene	8.096	c1ccc2c(c1)C(=C(C2)CCN(C)C)[C@H](c1ccccn1)C
nandrolone decanoate	7.604	[C@H]12[C@H]3[C@@H]([C@@H]4C(=CC(=O)CC4)CC3)CC[C@@]1([C@H](CC2)OC(=O)CCCCCCCCC)C
(1R,2S,6R,7S)-4-{[(1R,2R)-2-{[4-(1,2-benzothiazol-3-yl)piperazin-1-yl]methyl}cyclohexyl]methyl}-4-azatricyclo[5.2.1.0^{2,6}]decane-3,5-dione	7.921	C1[C@H]([C@@H]([C@H]([C@H]1O)C/C=C\CCCC(=O)OC(C)C)/C=C/C(COc1ccccc1)(F)F)O
vismodegib	7.649	c1cccc(n1)c1cc(ccc1Cl)NC(=O)c1ccc(cc1Cl)S(=O)(=O)C
pitavastatin	7.425	c1ccc2c(c1)c(c1ccc(cc1)F)c(/C=C/[C@H](C[C@H](CC(=O)O)O)O)c(C1CC1)n2
rilpivirine	6.66	c1(cc(c(c(c1)C)Nc1nc(ncc1)Nc1ccc(cc1)C#N)C)/C=C/C#N
crizotinib	7.352	Clc1ccc(F)c(c1[C@@H](C)Oc1cc(cnc1N)c1cnn(c1)C1CCNCC1)Cl
(3R)-3-cyclopentyl-3-(4-{7H-pyrrolo[2,3-d]pyrimidin-4-yl}pyrazol-1-yl)propanenitrile	5.042	c1c[nH]c2c1c(ncn2)c1cnn(c1)[C@H](CC#N)C1CCCC1
ethyl eicosapentaenoate	6.964	C(=C\C/C=C\C/C=C\C/C=C\C/C=C\CCCC(=O)OCC)\CC
mirabegron	6.36	c1cccc(c1)[C@H](CNCCc1ccc(cc1)NC(=O)Cc1csc(n1)N)O
regorafenib	7.389	c1(ccc(cc1C(F)(F)F)NC(=O)Nc1c(cc(cc1)Oc1ccnc(c1)C(=O)NC)F)Cl
{[5-(4-bromophenyl)-6-{2-[(5-bromopyrimidin-2-	6.559	C(CNS(=O)(=O)Nc1ncnc(c1c1ccc(cc1)Br)OCCOc1ncc(cn1)Br)C

yl)oxy]ethoxy}pyrimidin-4-yl]sulfamoyl}(propyl)amine		
4-[(1S,2R)-1-hydroxy-2-{[(2R)-1-phenoxypropan-2-yl]amino}propyl]phenol	6.344	C[C@H](COc1ccccc1)N[C@H](C)[C@@H](O)c1ccc(O)cc1
iotroxic acid	6.734	c12c(c[nH]c1cccc2)CCN1CCC(CC1)NC(=O)c1ccccc1
ifenprodil	7.088	C[C@@H]([C@@H](c1ccc(cc1)O)O)N1CC[C@@H](CC1)Cc1ccccc1
fenbufen	6.989	c1ccc(cc1)c1ccc(cc1)C(=O)CCC(=O)O
bicol	6.023	CC(=O)Oc1ccc(cc1)C(c1ccc(cc1)OC(=O)C)c1ccccn1
(2R,3R)-2-(2,4-difluorophenyl)-3-(4-methylidenepiperidin-1-yl)-1-(1,2,4-triazol-1-yl)butan-2-ol	6.977	c1cc(cc(c1[C@]([C@H](N1CCC(=C)CC1)C)(Cn1cncn1)O)F)F
6-hydroxy-8-[(1R)-1-hydroxy-2-{[1-(4-methoxyphenyl)-2-methylpropan-2-yl]amino}ethyl]-2,4-dihydro-1,4-benzoxazin-3-one	7.219	C1(=O)Nc2c(OC1)c(cc(c2)O)[C@H](CNC(Cc1ccc(cc1)OC)(C)C)O
tantum	6.852	CN(C)CCCOc1nn(Cc2ccccc2)c2ccccc12
eslicarbazepine acetate	5.493	O([C@@H]1c2c(N(c3c(C1)cccc3)C(=O)N)cccc2)C(=O)C
zucapsaicin	6.691	CC(C)/C=C\CCCCC(=O)NCc1ccc(c(c1)OC)O
(1S,2R,10R,11S,14S,15S)-14-acetyl-2,8,14,15-tetramethyltetracyclo[8.7.0.0^{2,7}.0^{11,15}]heptadeca-6,8-dien-5-one	8.238	C[C@]12[C@H]([C@H]3[C@H](CC2)[C@]2(C)C(=CC(=O)CC2)C(=C3)C)CC[C@@]1(C(=O)C)C
4-[(E)-2-[4-(2-{2-[2-(Ã,Â¹Ã¢?Â¸F)fluoroethoxy]ethoxy}ethoxy)phenyl]ethenyl]-N-methylaniline	6.395	c1(ccc(cc1)/C=C/c1ccc(cc1)OCCOCCOCC[18F])NC
4-[(E)-2-[6-(2-{2-[2-(Ã,Â¹Ã¢?Â¸F)fluoroethoxy]ethoxy}ethoxy)pyridin-3-yl]ethenyl]-N-methylaniline	5.797	[18F]CCOCCOCCOc1ncc(/C=C/c2ccc(NC)cc2)cc1
2-[3-(Ã,Â¹Ã¢?Â¸F)fluoro-4-(methylamino)phenyl]-1,3-benzothiazol-6-ol	5.181	c1c(ccc(c1[18F])NC)c1sc2c(n1)ccc(c2)O
moxisylyte	7.27	c1(c(cc(c(c1)C)OC(=O)C)C(C)C)OCCN(C)C
tolfenamic acid	6.753	Cc1c(cccc1Nc1ccccc1C(=O)O)Cl
3-{6-[1-(2,2-difluoro-1,3-benzodioxol-5-yl)cyclopropaneamido]-3-methylpyridin-2-yl}benzoic acid	7.954	O=C(O)c1cccc(c2nc(NC(=O)C3(c4ccc5OC(F)(F)Oc5c4)CC3)ccc2C)c1
propoxycaine	5.827	c1(c(cc(cc1)N)OCCC)C(=O)OCCN(CC)CC

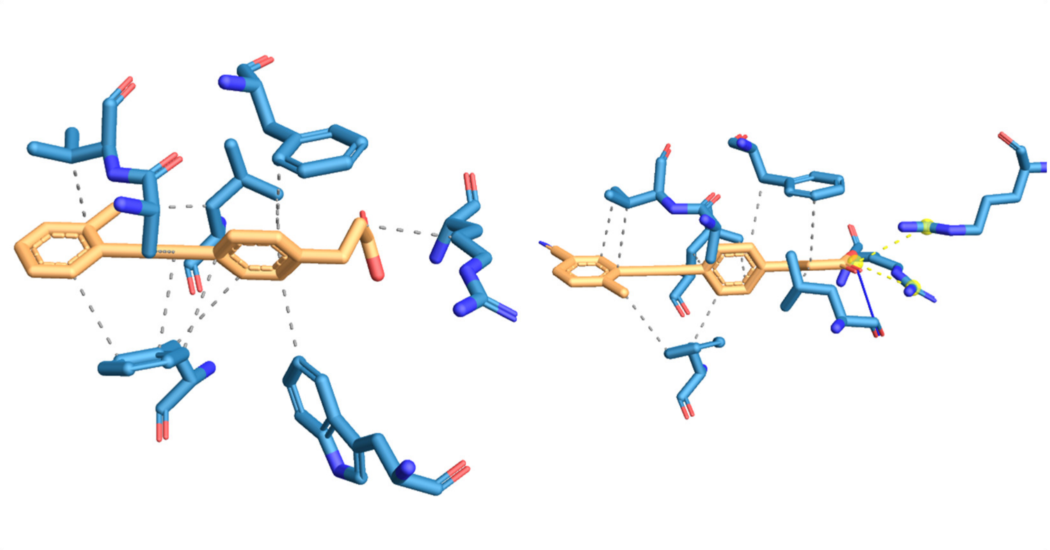

(**a**) (**b**)

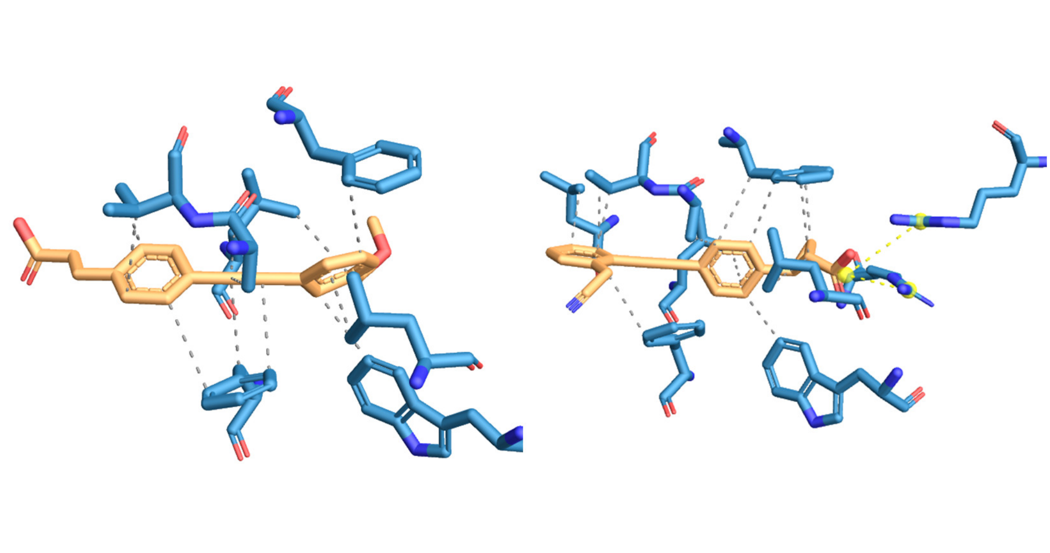

(**c**) (**d**)

Figure S2. 3D graphic of the interaction between FFA1 and compound (**a**) 15, (**b**) 91, (**c**) 92, and (**d**) 93

Table S8. Docking scores (DS, kcal/mol) for the 26 compounds derived from the screening databases.

DB ID	Name	DS	Database	DB ID	Compound	DS	Database
DB00837	Progabide	-7.0	DrugBank 5.1.7	DB11591	Bilastine	-9.1	DrugBank 5.1.7
DB00870	Suprofen	-8.1	DrugBank 5.1.7	DB11672	Curcumin	-8.8	DrugBank 5.1.7
DB00913	Anileridine	-9.0	DrugBank 5.1.7	DB13279	Carbocromen	-7.0	DrugBank 5.1.7
DB00963	Bromfenac	-8.5	DrugBank 5.1.7	DB13873	Fenofibric acid	-9.2	DrugBank 5.1.7
DB01138	Sulfinpyrazone	-9.5	DrugBank 5.1.7	DB00328	Indomethacin	-6.7	DrugBank 5.1.7
DB01395	Drospirenone	-8.0	DrugBank 5.1.7	DB00349	Clobazam	-7.0	DrugBank 5.1.7
DB01588	Prazepam	-6.9	DrugBank 5.1.7	DB00351	Megestrol acetate	-7.1	DrugBank 5.1.7
DB01608	Periciazine	-7.0	DrugBank 5.1.7	DB00425	Zolpidem	-7.5	DrugBank 5.1.7
DB04824	Phenolphthalein	-8.2	DrugBank 5.1.7	DB00715	Paroxetine	-7.0	DrugBank 5.1.7
DB04890	Bepotastine	-5.9	DrugBank 5.1.7	DB00768	Olopatadine	-6.4	DrugBank 5.1.7
DB05039	Indacaterol	-10.3	DrugBank 5.1.7	DB00829	Diazepam	-7.2	DrugBank 5.1.7
DB00186	Lorazepam	-7.3	DrugBank 5.1.7	DB08801	Dimetindene	-6.8	DrugBank 5.1.7
DB06209	Prasugrel	-6.3	DrugBank 5.1.7	-	Nuciferine	-6.0	DiaNat

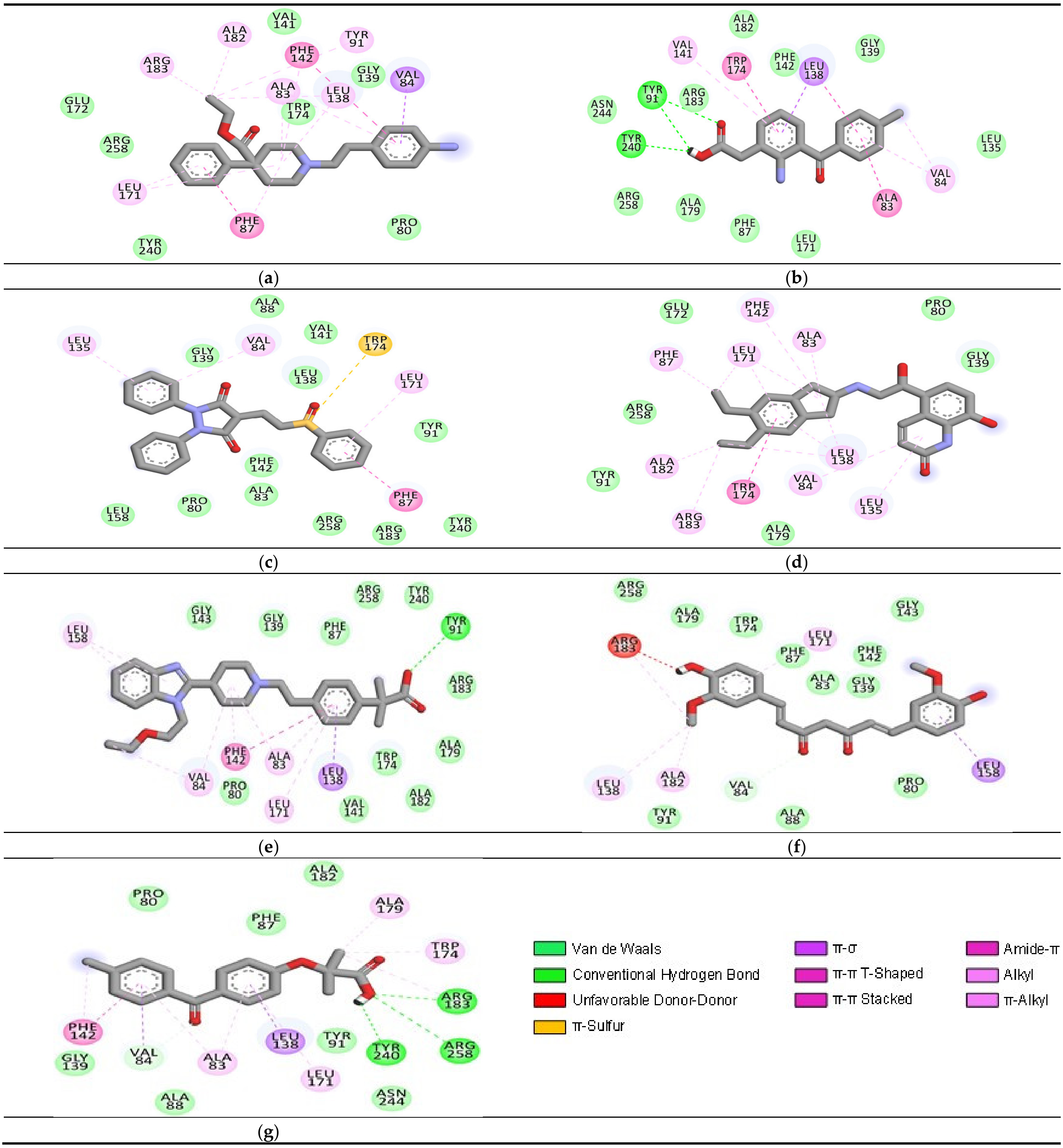

Figure S3. 2D graphic of the interaction between FFA1 and (**a**) anileridine, (**b**) bromfenac, (**c**) sulfinpyrazone, (**d**) indacaterol, (**e**) bilastine, (**f**)curcumin, and (**g**) fenofibric acid.

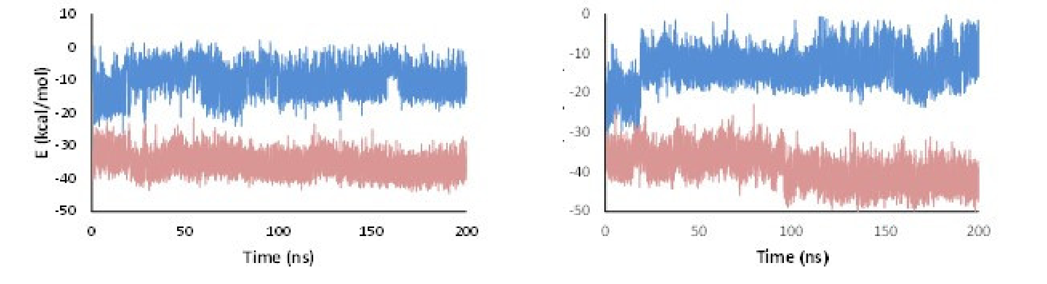

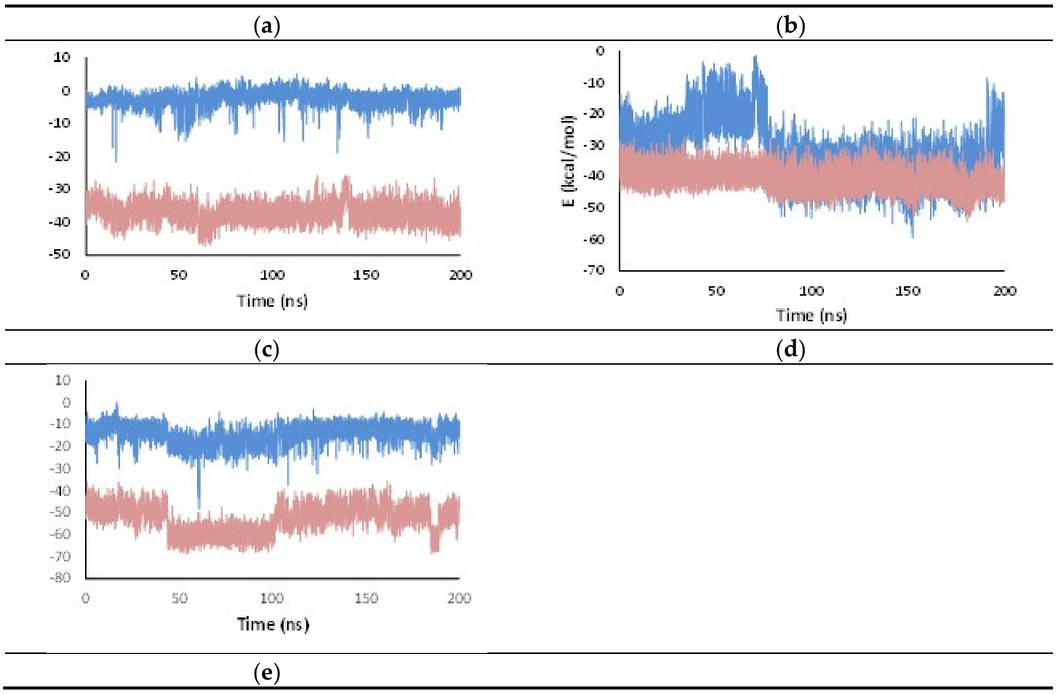

(a) (b) (c) (d) (e)

Figure S4. Coulomb (blue) and Lennard-Jones (red) interaction energies between FFA1 and compound 15 (**a**), 91 (**b**), 92 (**c**), 93 (**d**), and TAK-875 (**e**) during the 200 ns simulation.

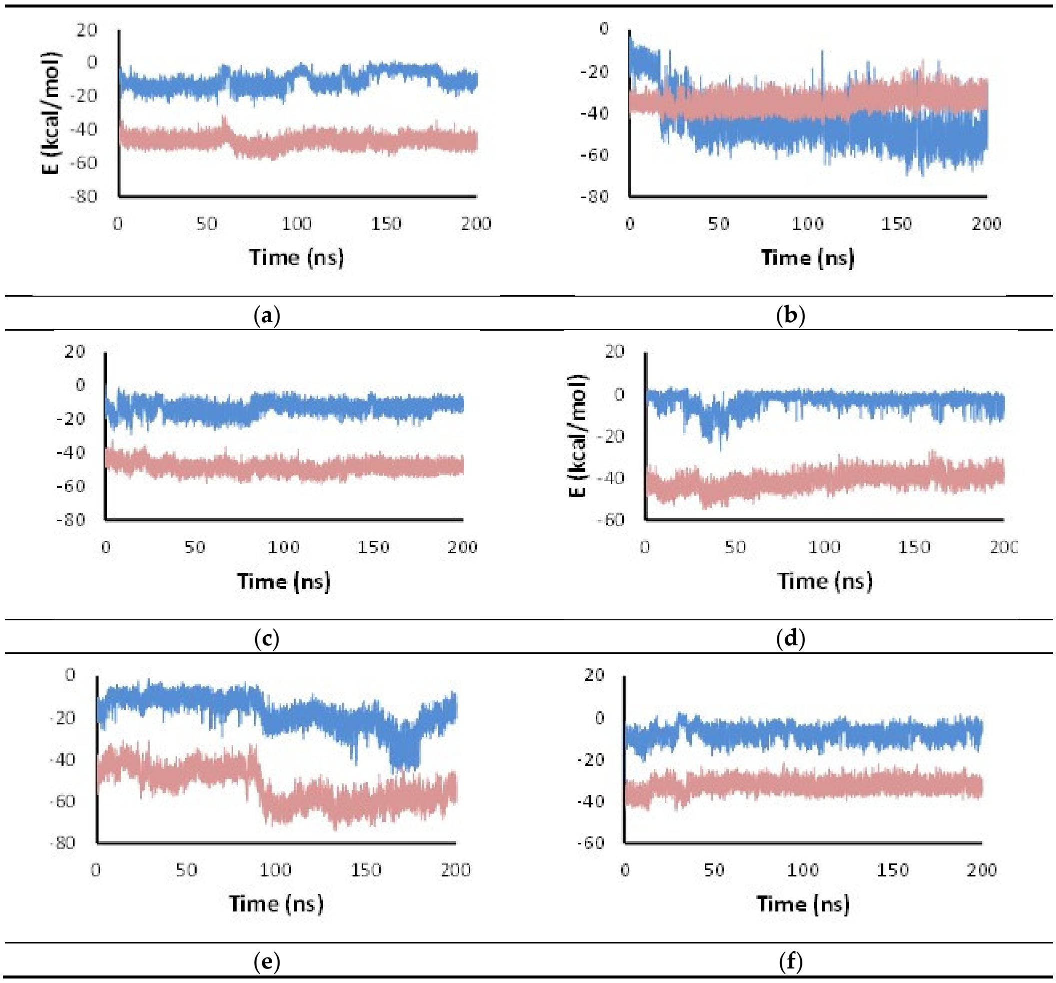

(a) (b) (c) (d) (e) (f)

Figure S5. Coulomb (blue) and Lennard-Jones (red) interaction energies between FFA1 and (**a**) anileridine, (**b**) bromfenac, (**c**) sulfinpyrazone, (**d**) indacaterol, (**e**) bilastine, and (**f**) fenofibric acid during the 200 ns simulation.
